# Salivary Gland Tumors in Iran: A Systematic Review of 2870 Cases Based on the New WHO Classification

**DOI:** 10.30699/IJP.2023.559121.2942

**Published:** 2023-03-23

**Authors:** Sahar Assar, Sepideh Assar, Heidar- Ali Mardanifard, Zohreh Jaafari-Ashkavandi

**Affiliations:** 1 School of Dentistry, Shiraz University of Medical Sciences, Shiraz, Iran; 2 Department of Mathematics, Yasouj University, Yasouj, Iran; 3 Department of Oral and Maxillofacial Pathology, School of Dentistry, Shiraz University of Medical Sciences, Shiraz, Iran

**Keywords:** Adenoid cystic carcinoma, Classification, Mucoepidermoid carcinoma, Neoplasms, Pleomorphic adenoma, Prevalence

## Abstract

**Background & Objective:**

There is no consensus on the prevalence of salivary gland tumors (SGTs) in Iran. Thus, we systematically reviewed the literature about the prevalence of SGTs in Iran and applied the last world health organization (WHO) classification.

**Methods:**

The systematic literature search was performed in EMBASE, Scopus, PubMed MEDLINE, Google Scholar, Scientific Information Database (SID), and Magiran; we searched for "salivary gland," "tumor," "prevalence," and "Iran" until 1 March 2021. The studies included were written in the English and Farsi languages. The weighted mean prevalence of SGTs was calculated as prevalence (%) * (N/the sum of all N). We used the unpaired Two-sample T-test to compare the weighted means.

**Results:**

A total of 17 studies, including 2870 patients, were selected for the data synthesis. The weighted mean prevalence of benign and malignant tumors was 66% (95% CI: 59-73) and 34% (95% CI: 27-41), respectively. The patients' mean age was reported in 10 out of the 17 studies. The weighted mean age of the patients was 40 (95% CI: 37-42) and 49 (95% CI: 43-55) years for benign and malignant tumors, respectively (*P*=0.01). Pleomorphic adenoma (PA), followed by Warthin's tumor (WT), was the most prevalent benign tumor. Moreover, the most common malignant tumors were mucoepidermoid carcinoma (MEC) and adenoid cystic carcinoma (AdCC).

**Conclusion:**

More than one-third of SGTs in Iran were malignant, which is higher than the reports from Middle Eastern countries. Information about risk factors and the burden of SGTs in Iran is insufficient. Thus, further well-designed longitudinal studies are warranted.

## Introduction

Salivary gland tumors (SGTs) are uncommon lesions that account for three to six percent of head and neck cancers (1–4). With an annual incidence rate of 0.4 to 13.5 per 100,000 population, SGTs are mostly benign and are usually found in parotid and minor salivary glands (1,5,6). 

The most common tumor of salivary glands is pleomorphic adenoma (PA), a benign, slow-growing, and painless tumor usually found in the parotid glands (7). In 2017, the world health organization (WHO) reclassified primary lesions of the salivary glands (8). New entities such as mammary analog secretory carcinoma, sclerosing polycystic adenosis, and intercalated duct hyperplasia were introduced. In contrast, some previous entities were compressed into broader classifications, and specific grading was removed from the titles (9). Another WHO classification was presented in 2022 (10). This edition (10) introduced some new rare entities, including sclerosing polycystic adenoma, keratocystoma, intercalated duct adenoma, and striated duct adenoma among the benign neoplasms; also, microsecretory adenocarcinoma and sclerosing microcystic adenocarcinoma were introduced as the new malignant entities. Cribriform adenocarcinoma of salivary gland origin (CASG) is now a subtype of polymorphous adenocarcinoma (PAC) (10). Moreover, the new classification has defined some subdivisions according to molecular studies and biomarkers (10). 

The epidemiology of SGTs is a function of genetics and environmental factors that vary according to the geographic region. It has been reported that SGTs are not common in Iran, and Iranian patients with malignant SGTs are younger with a different pattern of involvement than in other countries (11). There is no consensus on the prevalence of SGTs in Iran. Most of our knowledge comes from small descriptive studies (12,13), and the practice is according to the reports from other countries. Applying the last WHO classification, we systematically reviewed the literature on the prevalence of SGTs in Iran.

## Material and Methods

**Protocol, Registration, and Eligibility Criteria**

This systematic review was conducted according to the Preferred Reporting Items for Systematic Reviews and Meta-Analyses (PRISMA) (14). We designed the question according to the PICOS process (15) and registered the protocol in the International Prospective Register of Systematic Reviews (PROSPERO) under the registration number CRD42021258570. 

We included observational studies, randomized controlled trials, and non-randomized studies that investigated histopathologically confirmed cases of SGTs. Studies in the English or Farsi languages were included, with no limitation on age and gender. Reviews, case reports, book chapters, letters, conference abstracts, personal opinions, and studies without access to the full text were excluded. 

**Information Sources and Search Strategy**

We searched Google Scholar, Scientific Information Database (SID), and Magiran to find articles published in Persian. All the scientific articles in Farsi have an English abstract and English keywords. Therefore, it is possible to identify such papers using an English keyword combination in local databases/repositories such as SID and Magiran. We identified the English literature through EMBASE, Scopus, and PubMed MEDLINE. All the databases were searched until 1 March 2021. As a complementary search, we reviewed the reference list of the relevant studies. 

The following combination of the free-text terms was used on PubMed MEDLINE:

"(mucoepidermoid carcinoma OR pleomorphic adenoma OR Adenoid cystic carcinoma OR salivary gland) AND (tumor OR cancer) AND (incidence OR prevalence OR epidemiology OR rate) AND (Iran)." 

Although the combination rules could vary, we used the same terms in other databases/repositories (Appendix).

**Study Selection, Data Collection Process, and Measurements**

**Fig. 1:**
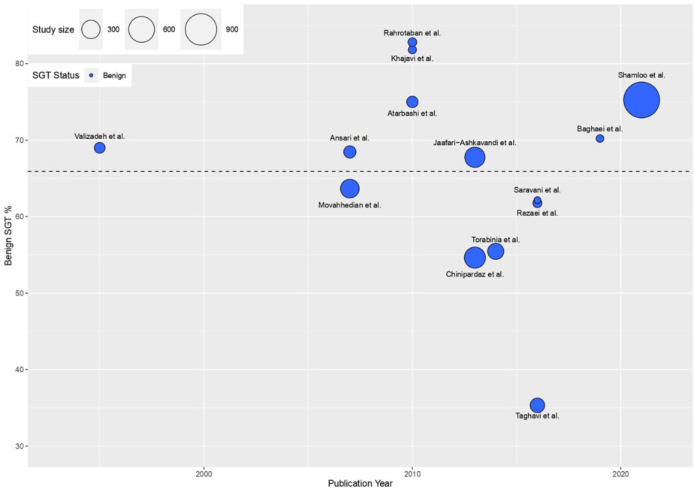
The visual presentation of the prevalence of benign SGTs in all the included studies. The weighted mean prevalence of benign SGTs was 66% (95% CI: 59-73) (Horizontal black dotted line)

**Fig. 2:**
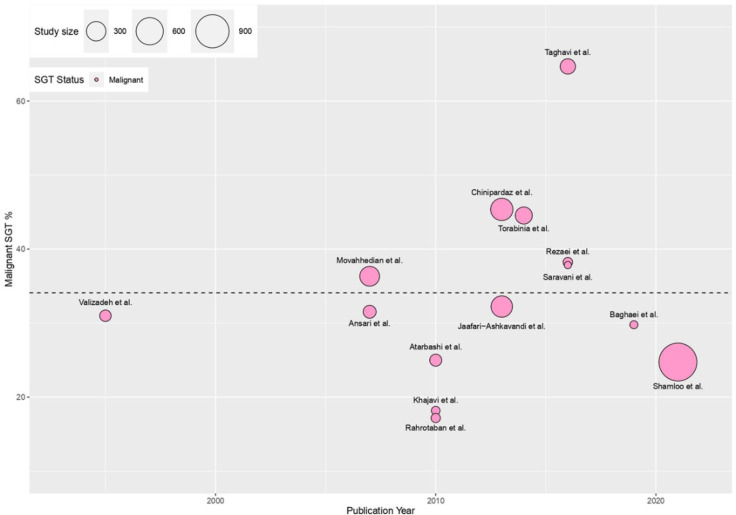
The visual presentation of the prevalence of malignant SGTs in all the included studies. The weighted mean prevalence of malignant SGTs was 34% (95% CI: 27-41) (Horizontal black dotted line).

Two independent researchers screened the titles and abstracts and excluded the papers that did not meet the inclusion criteria. Afterward, the full texts were reviewed, and the same two researchers extracted data. A third researcher resolved disagreements. For each study, the following data were extracted: type of study, publication year, the prevalence of SGTs, location of the SGTs, age, and gender. Data regarding the type of tumors were arranged according to the last WHO classification (10). The weighted mean prevalence of SGTs was calculated as prevalence (%) * (N/the sum of all N). Weighted scatterplots (bubble plots) were created using the ggplot2 data visualization package **(**Figures 1 and 2**)**. Due to the risk of selective bias, three studies that only included malignant SGTs (16–18) were excluded from calculations for weighted mean prevalence. We used the unpaired Two-sample T-test to compare the weighted means. All analyses were conducted in R, and *P*≤0.05 was considered statistically significant. 

**Risk of Bias Assessment of the Included Studies**

The Joanna Briggs Institute's (JBI's) critical appraisal checklist (version 2017) for prevalence studies (19) was used to assess the risk of bias. Among the nine domains of this checklist, "Sufficient coverage of the identified sample" and "Adequate response rate" were not applicable for the selected studies. Two independent researchers defined the risk of bias, and the third researcher solved disagreements. High, moderate, and low risk of bias were defined as ≤49%, 50-69%, and ≥70% positive answers (Yes) to the signaling question, respectively**.**

## Results

**Fig. 3:**
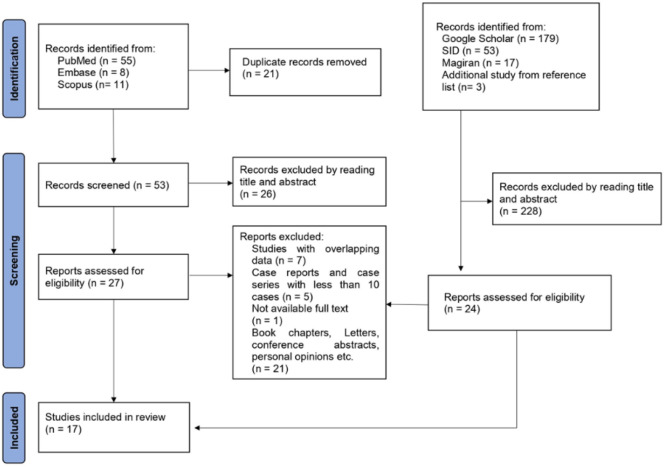
The study selection flowchart adapted from PRISMA (44)

Seventeen of the 323 records were suitable for data extraction (Figure 3), which resulted in the identification of 2870 SGTs, including 2003 and 867 benign and malignant tumors. A summary of the included studies is displayed in Table 1. Nine studies were conducted in central (11,18,20–26) Iran, four in western (27–30), two in eastern (17,31), and two in southern Iran (16,32). The patients' mean age was reported in 10 out of the 17 studies. The weighted mean age was 40 (95% CI: 37-42) years for patients with benign and 49 (95% CI: 43-55) years for patients with malignant tumors (*P*=0.01) **(**Supplementary Table 1). The weighted mean prevalence by gender for patients with benign tumors was 47% (95% CI: 40-55) and 53% (95% CI: 46-61) in males and females, respectively (*P*=0.214); also, for patients with malignant tumors, it was 45% (95% CI: 39-51) and 55% (95% CI: 49-61) in males and females, respectively (*P*=0.01). 

The reported prevalence of benign tumors ranged from 56- 81% (Table 1). The weighted mean prevalence of benign tumors was 66% (95% CI: 59-73) (Figure 1), and the weighted mean prevalence of malignant tumors was 34% (95% CI: 27-41) (Figure 2).

**New WHO Classification**

Based on the new classification, eleven cases of metastasizing PA were moved from the malignant category to the benign PA. Furthermore, inverted ductal and intraductal papilloma were merged as the ductal papilloma group. Two single cases of cystadenocarcinoma and papillary cystadeno-carcinoma were assumed as adenocarcinoma, not specific otherwise (NOS). Moreover, polymorphous low-grade adenocarcinoma was renamed to Poly-morphous adenocarcinoma. 

**Table 1:** Details of all studies

Ref	Author (publication year)	Geographic region, Number of the case (% in All or OMF)	Duration (Years)	Age, years Mean (range)	Female gender, %	Most common Malignancy
(27)	Ansari MH (2007)	Western, 130 (12% OMF)	20 (1984- 2003)	Total: 45 (13-77) Benign: 41(NR) Malignant: 47 (NR)	60%	**MEC**
(28)	Atarbashi Moghadam S (2010)	Western, 112 (NR)	11 (1997- 2008)	Total: NR (10-100) Benign: NR (20-40) Malignant: NR (70-79)	55%	**MEC**
(20)	Chinipardaz Z (2013)	Central, 399 (NR)	20 (1986- 2006)	Median (IQR) Total: 45 (27, 63) Benign: 40 (23, 56) Malignant: 52 (33, 71)	46%	**AdCC**
(16)	Hashemi PM (2007)	Southern, 70 (Malignant)	11 (1991- 2002)	Total: 50±18 (10-86) Benign: NR (NR) Malignant: NR (NR)	39%	**MEC**
(32)	Jaafari-Ashkavandi Z (2013)	Southern, 366 (3% OMF)	5 (2005- 2009)	Total: 42 ±17 (5-83) Benign: 38± 15 (NR) Malignant: 51±19 (NR)	49%	**AdCC**
(21)	Khajavi M (2010)	Central, 55 (NS)	10 (1996- 2005)	Total: 46± 13 (19-78) Benign: 42± 10 (NR) Malignant: 63± 14 (NR)	45%	**MEC**
(17)	Mohtasham N(2016)	Eastern, 98 (Malignant)	43 (1971-2013)	Total: 40(2-92) Benign: NR (NR) Malignant: NR (NR)	50%	**MEC**
(22)	Rahrotaban S(2010)	Central, 64 (0.65% All)	10 (1999-2009)	Total: 40± 17 (12-90) Benign: 39 ± 16 (NR) Malignant: 46± 18 (NR)	50%	**MEC**
(29)	Rezaei F (2016)	Western, 68 (1.1% All)	5 (2007-2012)	Total: NR (NR) Benign: 44± 17 (NR) Malignant: 57± 18 (NR)	38%	**AdCC**
(31)	Saravani S (2016)	Eastern, 37 (8.5% OMF)	11 (2002-2012)	Total: 32± 16 (NR) Benign: 31± 12 (NR) Malignant: 34 ± 21 (NR)	52%	**MEC**
(18)	Tabatabai S (2015)	Central, 81 ( Malignant)	11 (2001-2012)	Total: 53± 18 (10-100) Benign: NR (NR) Malignant: NR (NR)	57%	**AdCC**
(11)	Taghavi N (2016)	Central, 184 (0.4% All,3.03 OMF)	15 (2000-2015)	Total: NR ( 11-79) Benign: 41(NR) Malignant: 46 (NR)	60%	**MEC**
(23)	Torabinia N (2014)	Central, 229 (0.01 % All)	10 (2001- 2011)	Total: 46± 16 (9-80) Benign: 41 (NR) Malignant: 52 (NR)	51%	**MEC**
(24)	Valizadeh Y(1995)	Central, 103 (Minor)	20 (1972- 1992)	Total: NR (11-89) Benign: NR (NR) Malignant: NR (NR)	56%	**AdCC**
(25)	Movahedian (2007-8)	Central, 311 (NS)	5 (1998-2003)	Total: NR (NR) Benign: NR (30-50) Malignant: NR ( > 50)	45%	**MEC**
(30)	Baghaei F (2019)	Western, 47 (6.6% OMF)	11 (2006- 2017)	Total: NR (NR) Benign: NR (20-60) Malignant: NR (40-60)	43%	**MEC**
(26)	**Shamloo (2021)**	**Central, 1180 (0.86% All)**	**11(2005-2016)**	**Total: 43± 17 (2-90)** **Benign: 4± 16 (NR)** **Malignant: 46±17 (NR)**	**49%**	**AdCC**

OMF: Oral and maxillofacial; NR: Not reported; MEC: Mucoepidermoid carcinoma; AdCC: Adenoid cystic carcinoma; IQR: Interquartile range

**Location of the SGTs**

**Fig. 4:**
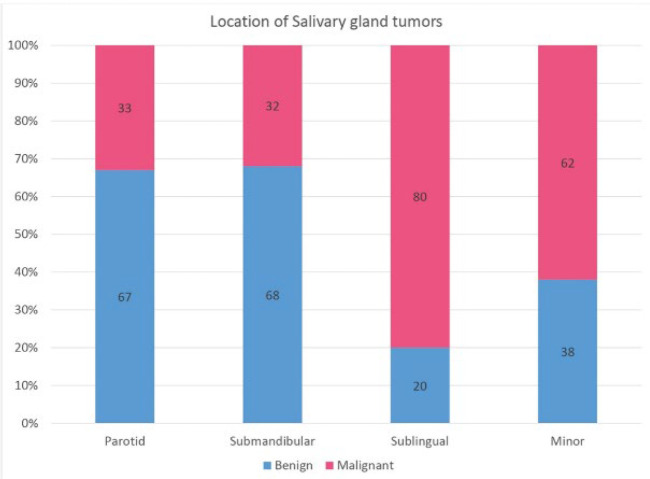
The prevalence of benign and malignant SGTs in each salivary gland

**Fig. 5:**
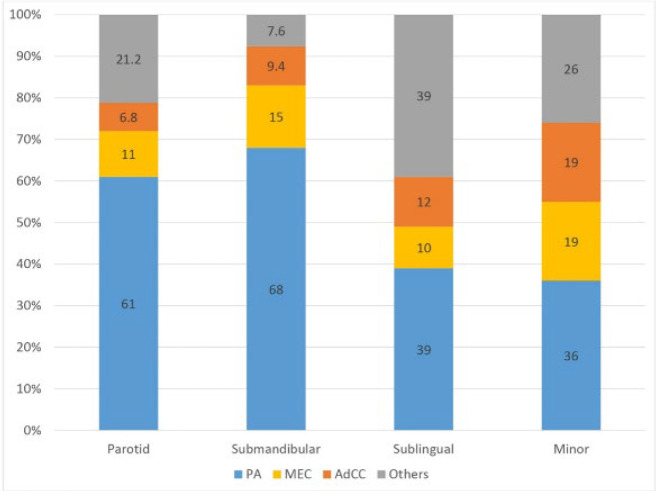
The prevalence of the most common SGTs in each salivary gland (in studies that presented the details) (11,18,20,23,26–28,32)

Tumor location was reported in 2166 out of 2870 SGTs. Most tumors were identified in the parotid (n=1455), followed by minor salivary glands (n=476), ,submandibular (n=199), and sublingual (n=36) glands.. The weighted mean prevalence of malignant tumors was 33% (95% CI: 19-47) and 32% (95% CI: 16-49) in the parotid and submandibular glands, respectively, while the weighted mean prevalence of malignant tumors was 80% (95% CI: -5.9-166) and 62% (95% CI: 48-76) in the sublingual and minor salivary glands, respectively (Figure 4). The prevalence of PA, MEC, and AdCC in each salivary gland is presented in Figure 5. 

**Tumor Types**

PA (92%, n = 1614) followed by Warthin's tumor (WT) (5.6%, n = 98) with a weighted mean age of 36 (95% CI: 32-41) and 54 (95% CI: 41-67) years were the most prevalent benign tumors, respectively. Furthermore, mucoepidermoid carcinoma (MEC) (35%, n = 282) and adenoid cystic carcinoma (AdCC) (34%, n = 270) with a weighted mean age of 44 (95% CI: 38-50) and 50 (95% CI: 47-53) years were the most frequent malignant tumors, respectively **(**Table 2**)**. The weighted mean prevalence in female patients with PA and WT was 56% (95% CI: 50-62) and 17% (95% CI: -15-49), respectively, whereas the weighted mean prevalence was 50% (95% CI: 43-58) and 57% (95% CI: 48-66) in female patients with MEC and AdCC, respectively.

**Table 2:** Baseline data of all benign and malignant tumors in the studies which reported the details of The patient's age and gender

Tumor	n (%)	Weighted mean age (95% CI)	M:F (n)
Benign Tumors	**1756 (100)**	**40 (37-42)**	871: 885
Pleomorphic adenoma	1614 (92)	36 (32-41)	**756: 858**
Warthin's tumor	98 (5.6)	54 (41-67)	**90: 8**
Monomorphic adenoma	26 (1.5)	54 (40-68)	**12: 14**
Oncocytoma	9 (0.5)	43 (-4.2-90.2)	**5: 4**
Ductal papilloma	9 (0.5)	NA	**8: 1**
Malignant tumors	**801 (100)**	**49 (43-55)**	403: 398
Mucoepidermoid carcinoma	282 (35)	44 (38-50)	**142: 140**
Adenoid cystic carcinoma	270 (34)	50 (47-53)	**126: 144**
Adenocarcinoma NOS	85 (11)	54 (45-63)	**45: 40**
Acinic cell carcinoma	47 (5.9)	42 (28-56)	**23: 24**
Carcinoma ex pleomorphic adenoma	34 (4.2)	49 (38-61)	**14: 20**
Squamous cell carcinoma	32 (4)	62 (38-66)	**24: 8**
Salivary duct carcinoma	14 (1.7)	60 (15-105)	**7: 7**
Polymorphous adenocarcinoma	14 (1.7)	58 (50-66)	**7: 7**
Epithelial myoepithelial carcinoma	11 (1.4)	NA	**7: 4**
Undifferentiated carcinoma	4 (0.5)	38 (26-50)	**3: 1**
Myoepitheial carcinoma	4 (0.5)	53 (3.3-102)	**3: 1**
Oncocytic adenocarcinoma	2 (0.3)	52	**1: 1**
Basal cell adenocarcinoma	1 (0.1)	55 (-197-307)	**1: 0**
Clear cell carcinoma	1 (0.1)	48 (23-73)	**0: 1**
Lymphoepihelial carcinoma	**1 (0.1)**	**NA**	**NA**

NA: No data available; NOS: Not otherwise specified

**Risk of Bias Within Studies**

Fifteen (88.2%) studies had a low risk of bias, mainly due to non-valid methods that were used for the identification or measurement of the condition in an unreliable way for all the included participants (Table 3). 

**Table 3:** The Joana Briggs Institute Critical Appraisal checklist domains used for the risk of assessment bias in the selected studies

Author Year	Appropriate sample to address the target population	Appropriate sampling way	Adequate sample size	A detailed description of the study subjects and settings	Sufficient coverage of the identified sample	Valid methods used for the identification of the condition	Measuring the condition in a standard, reliable way for all patients	Appropriate statistical analysis	Adequate response rate	Overall appraisal
Ansari M. 2007	Yes	No	No	Yes	Not applicable	Yes	Yes	Yes	Not applicable	**Low**
Atarbashi S. 2010	Yes	Yes	Unclear	Yes	Not applicable	Yes	Unclear	Yes	Not applicable	**Low**
Chinipardaz Z. 2012	Yes	Yes	Yes	Yes	Not applicable	Yes	Yes	Yes	Not applicable	**Low**
Hashemipour M. 2007	Yes	Yes	Yes	Yes	Not applicable	Unclear	Unclear	Yes	Not applicable	**Low**
Jaafari-Ashkavandi Z. 2014	Yes	Yes	Yes	Yes	Not applicable	Yes	Yes	Yes	Not applicable	**Low**
Khajavi M. 2010	Yes	No	Unclear	Yes	Not applicable	Yes	Yes	Yes	Not applicable	**Low**
Mohtasham N. 2015	Yes	Yes	Yes	No	Not applicable	Yes	Yes	Yes	Not applicable	**Low**
Rahrotaban S. 2010	Yes	Yes	Yes	No	Not applicable	Yes	Yes	Yes	Not applicable	**Low**
Rezaei F. 2016	Yes	Yes	Yes	Yes	Not applicable	Yes	Yes	Yes	Not applicable	**Low**
Saravani Sh. 2016	Yes	Yes	No	Yes	Not applicable	Yes	Yes	Yes	Not applicable	**Low**
Tabatabai H. 2015	Yes	Yes	Yes	Yes	Not applicable	Yes	Yes	Yes	Not applicable	**Low**
Taghavi N. 2015	Yes	Yes	Yes	Yes	Not applicable	Unclear	Unclear	Yes	Not applicable	**Low**
Torabinia N. 2013	Yes	Yes	Yes	Yes	Not applicable	Yes	Yes	Yes	Not applicable	**Low**
Valizadeh Y. 1995	Yes	No	Unclear	No	Not applicable	Unclear	Unclear	Yes	Not applicable	**High**
Movahedian B. 2007	Yes	Yes	No	Yes	Not applicable	Unclear	Unclear	Yes	Not applicable	**Moderate**
Baghaei F. 2019	Yes	Yes	Yes	Yes	Not applicable	Unclear	Unclear	Yes	Not applicable	**Low**
Shamloo N. 2020	**Yes**	**Yes**	**Yes**	**Yes**	**Not applicable**	**Yes**	**Yes**	**Yes**	**Not applicable**	**Low**

## Discussion

This systematic review aimed to investigate the prevalence of SGTs in Iran. About two-thirds of SGTs were benign, and the majority of the included patients were middle-aged women. Furthermore, the patients with malignant tumors were significantly older than those with benign tumors. 

The benign tumors accounted for 66% of SGTs, with the ratio of benign to malignant tumors being 1.9:1. In reports from other Middle Eastern countries such as Iraq (4.7:1), United Arab Emirates (2.8:1), Jordan (2.3:1), and Turkey (2.2:1), the prevalence of malignant tumors was lower than Iran (33–36), while in Nigeria (1: 2.5), Brazil (1.4:1), and China (1.8:1) this ratio is higher (5,37,38). Among the included studies in this systematic review, only one study (11) showed a higher prevalence of malignant tumors and with the ratio of benign to malignant tumors being 1:1.8, which is consistent with a report from Nigeria (1:2.5) (38) and could be due to bias in sampling or selection of cases. 

In our study, the most frequent benign tumors were PA and WT, and the most frequent malignant tumors were MEC and AdCC, respectively. This finding contrasts previous studies (18,20,24,26,29,32) that reported AdCC as the most prevalent malignant tumor. Following our results, some reports from United Arab Emirates, Nigeria, China, Brazil, and Mexico, showed a higher prevalence of MEC compared to AdCC (5,34,37–39). In contrast, in reports from the Netherlands, Poland, and Japan, AdCC was more common (6,40,41). Some of these studies (6,38,39,41) were carried out in a single center or only in a small country region, and geographical and ethnic variations may justify the differences. Studies with large sample sizes reported similar results to ours, and MEC and AdCC composed more than half of the malignant SGTs (5,37–39,41). 

In line with some other reports, we found a high prevalence of adenocarcinoma (NOS) (11% of all malignant tumors) (5,40,42). In the new classification, adenocarcinoma NOS was broadened to include rare tumors such as papillary cystadenocarcinoma and mucinous carcinoma (3). With the help of new molecular diagnostic approaches, some of these tumors might be classified into other groups. 

In this study, the mean age of the patients with benign and malignant tumors was 40 and 49 years, respectively. Consistent with this result, previous studies (5,37,43) showed that benign SGTs usually occur at younger ages (mostly the fourth and fifth decades of life), while malignant tumors are mainly reported in the fifth and sixth decades. Moreover, in the current study, patients with PA were younger than those with WT. However, patients with MEC were younger than those with AdCC. These findings are in the same line as those reported from Poland and Brazil (6,37). 

In our study, SGTs were more common in females. Reports from Iraq, China, Poland, and Brazil are consistent with this result (5,6,33,37); however, this finding is in contrast with the reports from Turkey, the Netherlands, and Japan, in which SGTs are more commonly reported in males (36,39,40). 

Most SGTs were identified in major salivary glands, with parotid as the most prevalent location. Most of the tumors in the parotid and submandibular glands were benign, whereas in sublingual and minor salivary glands, malignant tumors were more common. These findings are in agreement with previous studies (5,6,41,43). Moreover, in the current study, AdCC and MEC were equally prevalent in minor salivary glands. Reports from Brazil, China, and Mexico showed a higher prevalence of AdCC in minor salivary glands (5,37,39); in contrast, MEC was more common in studies from Poland, Japan, and Nigeria (6,38,^41^).

This systematic review had some limitations; the new WHO classification introduced some new tumors that could only be identified if the tumor morphology was re-evaluated and molecular diagnostic methods were considered. We could reclassify the tumors only based on the previous diagnoses; however, researchers in Japan and Mexico (39,41) could reclassify the SGTs via re-evaluation of the tumors and found that some polymorphous adenocarcinomas, secretory car-cinomas, and cribriform adenocarcinoma of minor salivary glands have been previously misdiagnosed as acinic cell adenocarcinoma and carcinoma ex PA or other tumors (38,39). However, Cunha et al. (39) reclassified 3.7% of all tumors based on morphological, molecular, and immunohistochemical re-evaluations. Most of the selected studies in this systematic review were related to an overlapping period, making it impossible to observe changes in tumor incidence over time. Finally, the differences among studies in data presentation (such as age), method of classification, period of study, and the institutes where the studies were conducted led to the loss of some cases in statistical analyses. However, due to a considerable number of cases, comprehensive and acceptable data were obtained. 

## Conclusion

This systematic review was carried out on the prevalence of SGTs in Iran. More than one-third of SGTs were malignant, which is higher than those reported from Middle Eastern countries. Most of the patients were in the fifth decade of life. The majority of the tumors in minor salivary glands were malignant, with an equal prevalence of AdCC and MEC. Our findings could be used by healthcare authorities and decision-makers to improve preventive strategies. Information about the risk factors and burden of SGTs in Iran is insufficient. Thus, further, well-designed longitudinal studies are warranted.

## Conflict of Interest

There are no conflicting interests.

## Funding

This research received no specific grant from any funding agency in the public, commercial, or not-for-profit sectors.
